# Knowledge and practices of personal hygiene among high school students in Kabul, Afghanistan: A cross-sectional study

**DOI:** 10.1097/MD.0000000000049492

**Published:** 2026-06-26

**Authors:** Hafizullah Nikzad, Bashir Ahmad Qudrati, Mohammad Ali Reshad, Shabir Ahmad Qudrati, Mursal Maulawizada

**Affiliations:** a Public Health Faculty, Kabul University of Medical Sciences, Kabul, Afghanistan; b Department of Sociology, Kabul Education University, Kabul, Afghanistan; c Department of Emergency and Trauma Surgery, Ali Abad Teaching Hospital, Kabul, Afghanistan.

**Keywords:** Afghanistan, behavioral barriers, personal hygiene, school students

## Abstract

Personal hygiene (PH) is a fundamental practice crucial for preventing communicable diseases and promoting overall health, especially among school-aged children. Despite global recognition of its importance, data on hygiene-related knowledge and behaviors among students in Afghanistan are scarce. This appears to be the first study in Afghanistan to specifically examine knowledge and practices of PH in this population. This study aimed to assess the level of knowledge and practices of PH among school students in Kabul, Afghanistan. A cross-sectional study was conducted from May to June 2025, involving 211 male students from Habibia High School, selected via systematic random sampling. Data were analyzed using descriptive statistics and chi-square tests. Significance was set at *P* < .05. Of 211 distributed questionnaires, 202 were fully completed (response rate: 95.7%). The mean age of the participants was 16.66 years, with a standard deviation of 1.38. Based on predefined scoring criteria, 56.4% of students demonstrated good knowledge, while 87.6% demonstrated good PH practices. Handwashing, toothbrushing, and nail trimming were widely practiced; however, only 29.2% reported using soap during handwashing, despite high awareness of its importance. Although 97% reported bathing regularly, daily bathing was limited to 30.7% in summer and only 5% in winter. Chi-square analysis showed no significant associations between hygiene knowledge/practices and sociodemographic factors, except for a significant difference in hygiene practices among 10th-grade students (*P* = .006). Overall, most sociodemographic characteristics were not significantly associated with students’ hygiene knowledge and practices. Although overall levels of hygiene knowledge and practice were satisfactory, gaps such as limited soap use, inconsistent bathing, and partial adherence to handwashing and nail trimming highlight the need for targeted school-based interventions. Strengthening hygiene education alongside improved access to sanitation resources may help sustain positive hygiene behaviors among students.

## 1. Introduction

Personal hygiene (PH) encompasses the practices that individuals adopt to maintain cleanliness and promote overall health and well-being. These practices include regular bathing, oral care, handwashing, grooming, and wearing clean clothing, all of which are essential for disease prevention and overall well-being.^[1–4]^ In many public health contexts, PH is recognized as a foundational and cost-effective strategy for reducing the spread of communicable diseases such as diarrhea, respiratory infections, skin infections, and helminthiasis.^[5,6]^

In low-income and developing countries, inadequate hygiene remains a pressing concern, contributing significantly to morbidity and mortality. Approximately 80% of common diseases in these regions are associated with poor hygiene practices, both at the individual and household levels.^[5–7]^ According to global health estimates, children collectively lose hundreds of millions of school days annually due to illnesses that stem from preventable hygiene-related conditions. Specifically, an estimated 443 million school days are lost each year because of waterborne diseases and sanitation issues.^[3,8]^ Diarrheal diseases alone result in more than 2 million deaths annually, with young children being particularly susceptible.^[6,7]^ Infectious diseases remain the leading cause of death in South Asia and sub-Saharan Africa, accounting for approximately 62% and 31% of total mortality, respectively – figures often exacerbated by poor hygiene and unsafe water sources.^[6,9]^

Children attending school are especially prone to hygiene-related health challenges due to limited awareness, lack of access to sanitary facilities, and insufficient hygiene education. Numerous studies have demonstrated that improved knowledge and hygiene practices among students can significantly reduce illness rates and school absenteeism and even enhance academic performance.^[6,10]^ Educational institutions play a fundamental role in shaping early health behaviors, offering an ideal environment for teaching lifelong hygiene habits through structured health education and teacher guidance.^[11,12]^ Teachers, often being the primary point of contact in educational settings, serve as influential role models in promoting hygienic behavior.

Despite global awareness of the importance of PH and substantial research conducted in various countries, data on this topic remain scarce in Afghanistan. Afghanistan has experienced decades of armed conflict, political instability, and economic hardship, all of which have substantially weakened its public health and educational infrastructure. In Kabul, rapid urban population growth, overcrowded classrooms, limited water, sanitation, and hygiene facilities in schools, and socioeconomic disparities may directly influence students’ hygiene behaviors and access to basic sanitary resources. Many public schools operate with constrained resources, and structured school-based hygiene promotion programs remain limited. These contextual challenges make it particularly important to generate local evidence on students’ hygiene knowledge and practices to inform feasible, context-sensitive public health interventions.

To date, no studies have systematically examined the knowledge and practices of PH among school students in this context. Considering the significant health challenges faced by Afghan children, including limited access to clean water and adequate sanitation facilities, this study aims to fill this research gap by assessing knowledge and practices of PH among school students in Kabul, Afghanistan. Filling this gap will provide essential baseline data to guide effective health education programs, inform public health policies, and ultimately contribute to reducing hygiene-related diseases in this vulnerable population.

## 2. Methods

### 2.1. Study design and setting

This cross-sectional study was conducted to assess the knowledge and practices of PH among school students. The study was carried out at Habibia High School, one of the oldest public secondary schools in Kabul, Afghanistan, enrolling male students in grades 10 to 12 during the 2025 academic year.

Habibia High School is a boys-only institution operating within the public education system of Kabul. Similar to other public schools in the city, access to sanitation infrastructure, including handwashing facilities, clean water, and hygiene supplies, may be variable. Data collection was conducted between May and June 2025.

### 2.2. Inclusion and exclusion criteria

The study included all male high school students enrolled at Habibia High School during the academic year of 2025 in grades 10 to 12 who were actively attending classes at the time of data collection. The school is a boys-only institution; therefore, female students were not eligible. Participants were required to provide verbal or written consent from their parents or legal guardians and agree to participate voluntarily.

Students were excluded if they were absent during the data collection period, declined to participate, or submitted incomplete questionnaires that could not be included in the analysis. A total of 9 students were excluded for submitting incomplete questionnaires, which accounted for a small proportion of nonparticipation.

### 2.3. Sample size and sampling method

The total number of high school students enrolled at Habibia High School in 2025 was 418. Using Epi Info software version 7.2.7, the minimum required sample size was calculated with a 95% confidence level, a 5% margin of error, and an assumed prevalence of 50% to maximize sample size in the absence of prior prevalence data. Based on these parameters, the calculated sample size was determined to be 202 students. An estimated 5% non-response was considered, resulting in an increased final sample size of 211 participants.

A systematic random sampling method was applied. The sampling interval (k) was calculated by dividing the total population (418) by the desired sample size (202), resulting in an interval of approximately 2. A simple random draw (balloting) was used to select a starting number between 1 and 2, after which every second student on the list was included until the required sample size was reached.

Potential sources of bias, including selection bias due to systematic sampling and information bias due to self-reported responses, were considered. Measures to minimize bias included random selection of the first participant, strict adherence to the sampling interval, pretesting and validation of the questionnaire, and assurances of confidentiality to encourage honest responses.

### 2.4. Data source and measurement

Based on previous studies conducted on PH,^[6,10,13,14]^ a structured questionnaire was developed and translated into the local language. The questionnaire was back-translated into English to ensure translation accuracy. Content validity was assessed by a panel of public health experts. A pilot study was conducted among 20 students to assess clarity and reliability. The internal consistency of the knowledge and practice sections was evaluated using Cronbach alpha (α = 0.79 for knowledge and α = 0.83 for practice).

The instrument consisted of 25 closed-ended, multiple-choice questions, divided into 3 sections. The first section contained 5 items related to students’ demographic information. The second section assessed knowledge of PH through 9 questions. The third section evaluated hygiene-related practices using 11 items. Each knowledge and practice question was scored as 1 point for a correct or appropriate answer and 0 points for incorrect or inappropriate responses. Knowledge and practice scores were treated as quantitative variables and categorized to facilitate interpretation and comparison.

The knowledge scores ranged from 0 to 9. Based on the total score, the students were categorized into 3 groups: poor knowledge (scores of 0–3), moderate knowledge (scores of 4–6), and good knowledge (scores of 7–9). Similarly, the practice scores ranged from 0 to 11. The participants were classified into 2 categories based on their total practice score: good practice (score ≥ 6) and poor practice (score < 6). These cutoffs were derived from the total possible scores and informed by previous similar studies. No clinical diagnostic criteria were applied, as the study was based solely on self-reported questionnaire data. The primary outcome variables of this study were students’ knowledge and practice regarding PH.

### 2.5. Data analysis

All statistical analyses were conducted using IBM Statistical Package for the Social Sciences Statistics version 27. Descriptive statistics, including frequencies and percentages, were used to summarize the categorical variables. The chi-square test was applied to assess associations between categorical variables. A *P* value of <.05 was considered statistically significant. The analysis was limited to descriptive and bivariate assessments; no multivariable regression was performed, and potential confounders or effect modifiers were not adjusted for due to the cross-sectional design and sample size. Subgroup analyses and examination of interactions between variables were not performed due to the cross-sectional study design and the limited sample size.

### 2.6. Ethics statement

Ethical approval was obtained from the Institutional Review Board of the Faculty of Public Health, Kabul University of Medical Sciences (Protocol ID: KUMS/PH/2025/190). Written informed consent was obtained from parents or legal guardians, and verbal assent was obtained from participating students before data collection. Participation was voluntary, and confidentiality was maintained by anonymizing all collected data. The study was conducted in accordance with the principles of the Declaration of Helsinki.

## 3. Results

### 3.1. Sociodemographic characteristics of participants

Of the 211 questionnaires distributed, 202 were fully completed and included in the final analysis, resulting in a response rate of 95.73%. The mean age of the participants was 16.66 years, with a standard deviation of 1.38. As shown in Table 1, the majority of students were younger than 17 years, 137 (67.8%). Most of the participants were enrolled in grade 10, 103 (51%). Regarding economic background, the majority of students reported having an average household economic status of 150 (74.3%).

**Table 1:** Demographic characteristics of the participants (N = 202).

Characteristics	Category	Frequency	%
Age group	<17	137	67.8
	≥17	65	32.2
Grade	10th	103	51
	11th	54	26.7
	12th	45	22.3
Economic situation	Poor	28	13.9
	Average	150	74.3
	Good	24	11.9
Father’s education level	Illiterate	154	76.2
	Secondary education	34	16.8
	Bachelor’s degree	10	5
	Master’s degree	4	2
Mother’s education level	Illiterate	189	93.5
	Secondary education	6	3
	Bachelor’s degree	4	2
	Master’s degree	3	1.5

Data are presented as frequencies and percentages to describe the sociodemographic characteristics of the participants.

The parental education levels of the participants were generally low. More than three-quarters of the students’ fathers were illiterate, 154 (76.2%), while smaller proportions had completed secondary education, 34 (16.8%); bachelor’s, 10 (5%); or master’s, 4 (2%). Similarly, most mothers were illiterate, 189 (93.5%), with only a few having completed secondary education, 6 (3%); bachelor’s, 4 (2%); or master’s, 3 (1.5%).

### 3.2. Knowledge of PH

**Figure 1.**
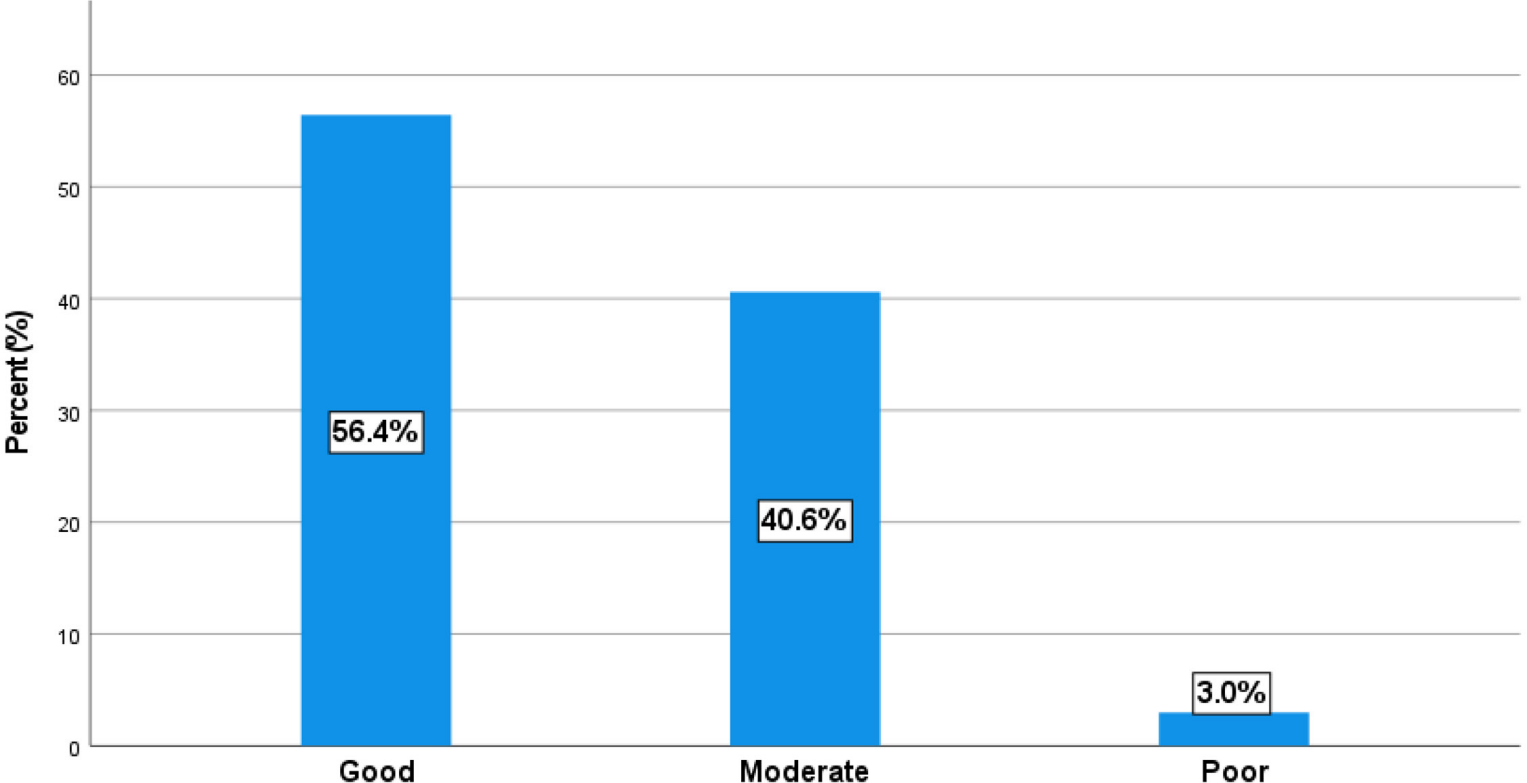
Proportion of students categorized by their level of knowledge about personal hygiene.

Regarding the level of knowledge about PH, the majority of students, 114 (56.4%), demonstrated good knowledge, 82 (40.6%) had moderate knowledge, and 6 (3%) had poor knowledge in this area (Fig. 1).

The assessment of students’ knowledge regarding PH indicated that 175 (86.6%) had heard of the term “personal hygiene.” Furthermore, 132 (65.3%) of students demonstrated a holistic understanding of PH, encompassing cleanliness, health maintenance, and disease prevention.

Regarding specific knowledge items related to hygiene behaviors, almost all students acknowledged the importance of handwashing, 197 (97.5%); brushing with toothpaste, 200 (99%); and daily bathing, 197 (97.5%). Similarly, a large majority, 200 (99%), believed that being clean and organized contributes to good health, while many, 194 (96%), recognized that washing hands with soap is more effective than using water alone.

Several perceived barriers to maintaining PH were reported. Laziness was the most cited barrier, 139 (68.8%), followed by lack of time, 62 (30.7%); lack of water, 47 (23.3%); inadequate education, 34 (16.8%); and 9 (4.5%) reported religious beliefs as a barrier. A detailed summary of participants’ knowledge is presented in Table 2.

**Table 2:** Students’ knowledge of personal hygiene.

Variables	Category	Frequency	%
Awareness of the term “personal hygiene”	Yes	175	86.6
	No	27	13.4
Understanding of the concept of personal hygiene	Cleanliness	46	22.8
	Maintaining health	20	9.9
	Disease prevention	4	2
	Holistic understanding	132	65.3
Awareness of the importance of handwashing	Yes	197	97.5
	No	5	2.5
Recognition of personal hygiene as essential for a healthy life	Yes	202	100
	No	0	0
Belief that being clean and organized contributes to good health	Yes	200	99
	No	2	1
Knowledge of the preventive role of brushing with toothpaste	Yes	200	99
	No	2	1
Awareness that handwashing with soap is more effective than using water alone	Yes	194	96
	No	8	4
Belief in necessity of daily bathing for health	Yes	197	97.5
	No	5	2.5
Perceived barriers to maintaining personal hygiene	Laziness	139	68.8
	Lack of time	62	30.7
	Lack of education	34	16.8
	Lack of water	47	23.3
	Religious beliefs	9	4.5

Data are presented as frequencies and percentages to describe students’ knowledge about personal hygiene.

### 3.3. Practice of PH

**Figure 2.**
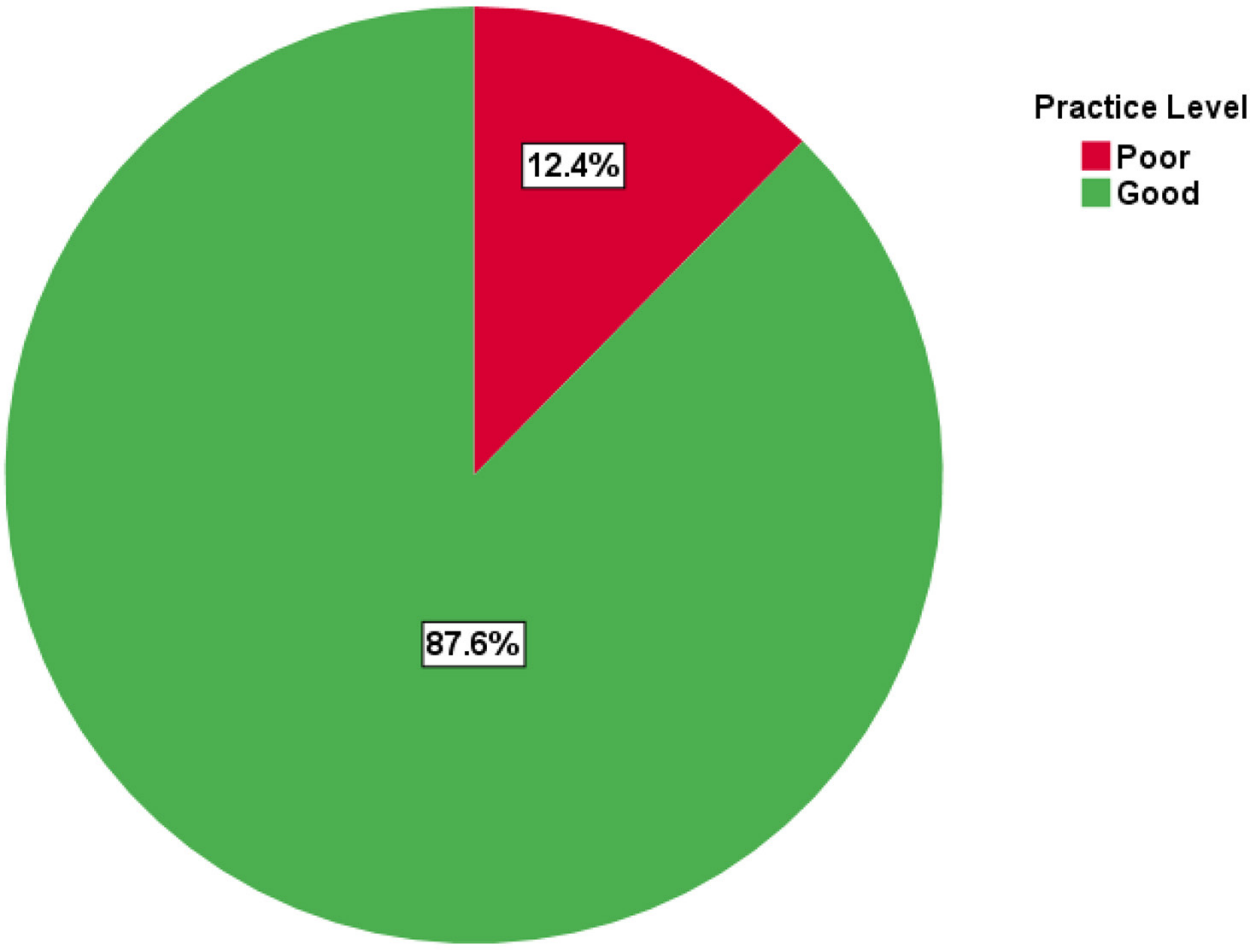
Proportion of students by level of adherence to personal hygiene practices, highlighting that most exhibit positive behaviors, while a smaller segment requires improvement.

Regarding the level of PH practices, the findings revealed that the majority of students, 177 (87.6%), exhibited good practice, while 25 (12.4%) reported poor practice. These findings indicate that most students reported engaging in appropriate PH behaviors. The detailed distribution is illustrated in Figure 2.

The findings related to students’ hygiene practices indicate a generally good adherence to PH routines. Most respondents reported taking regular baths, 196 (97%), and washing their hair regularly, 199 (98.5%). However, in summer, only 62 (30.7%) of students bathe daily, while the majority, 102 (50.5%), bathe twice a week. In winter, these numbers decrease, with only 10 (5%) of students bathing daily and 120 (59.4%) bathing once per week.

Concerning oral hygiene, 114 (56.4%) students reported brushing their teeth once daily, 35 (17.3%) twice daily, and 36 (17.8%) 3 times a day, while 17 (8.4%) reported never brushing daily. A large number, 185 (91.6%), reported washing their feet after school, while 17 (8.4%) reported doing so occasionally. Clothing change frequency varied by season; in summer, 21 (10.4%) reported changing clothes daily, whereas the majority, 113 (55.9%), reported changing clothes twice a week, and 68 (33.7%) once a week. In winter, 28 (13.9%) reported changing clothes daily, while 114 (56.4%) reported changing clothes once per week.

Regarding hand hygiene, all 202 participants (100%) reported washing hands before and after eating. However, 135 (66.8%) reported using water only, 59 (29.2%) used soap and water, and 8 (4%) used hand sanitizer. Most students, 192 (95%), reported covering their mouths when sneezing or coughing, and 201 (99.5%) reported trimming their nails regularly. A detailed summary of these practices is provided in Table 3.

**Table 3:** Students’ practices on personal hygiene.

Variables	Category	Frequency	%
Taking regular baths	Yes	196	97
	No	6	3
Washing hair regularly	Yes	199	98.5
	No	3	1.5
Frequency of bathing in summer	Every day	62	30.7
	Twice a week	102	50.5
	Once a week	36	17.8
	Once every 15 d	2	1
Frequency of bathing in winter	Every day	10	5
	Twice a week	58	28.7
	Once a week	120	59.4
	Once every 15 d	14	6.9
Daily toothbrushing frequency	Once	114	56.4
	Twice	35	17.3
	Three times	36	17.8
	Never	17	8.4
Washing feet after school	Yes	185	91.6
	No	0	0
	Sometimes	17	8.4
Frequency of changing clothes in summer	Daily	21	10.4
	Twice a week	113	55.9
	Once a week	68	33.7
Frequency of changing clothes in winter	Daily	28	13.9
	Twice a week	60	29.7
	Once a week	114	56.4
Handwashing before and after eating	Yes	202	100
	No	0	0
Method of handwashing before/after eating	Water only	135	66.8
	Soap and water	59	29.2
	Hand sanitizer	8	4
Covering mouth when sneezing/coughing	Yes	192	95
	No	10	5
Regular nail trimming	Yes	201	99.5
	No	1	0.5

Data are presented as frequencies and percentages to describe students’ practices regarding personal hygiene.

### 3.4. Association of sociodemographic factors with knowledge and practice of PH

The analysis revealed no statistically significant differences in knowledge level (*P* = .137) or hygiene practice (*P* = .070) across age groups. Students below 17 years had a higher proportion of good knowledge, 76 (66.7%), and good practice, 124 (70.1%), but these differences were not statistically significant.

Among grades, knowledge levels did not differ significantly (*P* = .149); however, hygiene practice was significantly higher among 10th-grade students, with 93 (52.5%) demonstrating good practice (*P* = .006) compared with other grades. The economic status of the family showed no significant effect on knowledge (*P* = .946) or practice (*P* = .959).

Father’s education level did not significantly influence knowledge (*P* = .105) or practice (*P* = .720). Among students with good knowledge, 86 (75.4%) had illiterate fathers. Similarly, 136 (76.8%) of students with good practice had illiterate fathers. Mother’s education level also showed no significant association with knowledge (*P* = .433) or practice (*P* = .447). Among students with good knowledge, 104 (91.2%) had illiterate mothers, while 166 (93.8%) of those with good practice had illiterate mothers. Overall, except for grade level in relation to hygiene practice, none of the examined sociodemographic variables showed a statistically significant association with knowledge or practice levels (Table 4).

**Table 4:** Association of sociodemographic factors with students’ knowledge and practices of personal hygiene.

Variables	Knowledge level					Practice level			
	Good	Moderate	Poor	Chi-square	*P* value	Good	Poor	Chi-square	*P* value
Age group									
< 17	76 (66.7%)	59 (72%)	2 (33.3%)	3.981	.137	124 (70.1%)	13 (52%)	3.273	.070
≥ 17	38 (33.3%)	23 (28%)	4 (66.7%)			53 (29.9%)	12 (48%)		
Grade									
10th	56 (49.2%)	46 (56.1%)	1 (16.7%)	6.754	.149	93 (52.5%)	10 (40%)	10.100	.006*
11th	29 (25.4%)	21 (25.6%)	4 (66.6%)			41 (23.2%)	13 (52%)		
12th	29 (25.4%)	15 (18.3%)	1 (16.7%)			43 (24.3%)	2 (8%)		
Economic situation									
Poor	14 (12.3%)	13 (15.8%)	1 (16.7%)	0.743	.946	25 (14.1%)	3 (12%)	0.083	.959
Average	86 (75.4%)	60 (73.2%)	4 (66.6%)			131 (74%)	19 (76%)		
Good	14 (12.3%)	9 (11%)	1 (16.7%)			21 (11.9%)	3 (12%)		
Father’s education level									
Illiterate	86 (75.4%)	63 (76.8%)	5 (83.3%)	10.510	.105	136 (76.8%)	18 (72%)	1.338	.720
Secondary education	19 (16.7%)	14 (17.1%)	1 (16.7%)			29 (16.4%)	5 (20%)		
Bachelor’s degree	9 (7.9%)	1 (1.2%)	0			8 (4.5%)	2 (8%)		
Master’s degree	0	4 (4.9%)	0			4 (2.3%)	0		
Mother’s education level									
Illiterate	104 (91.2%)	79 (96.3%)	6 (100%)	5.917	.433	166 (93.8%)	23 (92%)	2.658	.447
Secondary education	3 (2.7%)	3 (3.7%)	0			6 (3.4%)	0		
Bachelor’s degree	4 (3.5%)	0	0			3 (1.7%)	1 (4%)		
Master’s degree	3 (2.6%)	0	0			2 (1.1%)	1 (4%)		

All analyses were performed using the chi-square test and cross-tabulation. The significance level was set at *P* value <.05.

* Indicates statistical significance.

## 4. Discussion

This study examined the level of knowledge and practices of PH among school students in Kabul. The findings revealed that students’ knowledge levels were categorized into 3 groups: good (56.4%), moderate (40.6%), and poor (3%). These results are closely aligned with findings from studies conducted in Fiche Town, Ethiopia (59.2%),^[15]^ and Angolela, Ethiopia (52%).^[10]^ Moreover, the level of good knowledge reported in the present study is significantly higher than that observed in a study conducted in the Marko district.^[8]^ Conversely, it is lower than the findings from a study conducted in Nepal, where 88.5% of students demonstrated good knowledge.^[13]^ This variation may be attributed to differences in educational systems, the content of school curricula, the level of public awareness regarding PH, or the extent and effectiveness of school-based health education programs.

The findings of this study indicated that the vast majority of students (97.5%) were well aware of the importance of handwashing. This level of awareness is significantly higher than that reported in various other settings, including Marko District (23%),^[8]^ Fiche Town, Ethiopia (59.6%),^[15]^ Yirgalem Town (39.1%),^[7]^ Debark Town (52.2%),^[16]^ Arba Minch Town (22.3%),^[17]^ and Sabeta Town (32%).^[18]^ This discrepancy may be attributed to several factors, such as a stronger emphasis on PH education in Kabul schools, greater access to health information through media channels, broader public awareness campaigns, and the proactive role of teachers in promoting hygienic practices among students.

The findings revealed that 96% of students acknowledged that washing hands with soap is more effective than using water alone. However, only 29.2% reported using soap and water for handwashing before and after eating, while 66.8% reported using water only. This level of knowledge is substantially higher than that reported in studies from India (29.1%),^[19]^ Marko District (26.8%),^[8]^ the United Arab Emirates (71%),^[6]^ Bangladesh (71.6%),^[20]^ and Ethiopia (59.7%).^[15]^ Such variation may be attributed to increased public awareness in recent years regarding the role of soap in preventing infectious diseases, particularly in the aftermath of the COVID-19 pandemic, as well as the implementation of focused school-based education programs emphasizing the importance of using soap during handwashing in Kabul.

The present study found that a majority of students (56.4%) reported brushing their teeth once per day, while only 17.3% did so twice daily. These findings are comparable with those from a study conducted in Ethiopia, where 53.2% of students brushed once, and 27.5% brushed twice per day.^[15]^ In contrast, a study conducted in Saudi Arabia reported that 71.7% of students brushed their teeth twice daily,^[4]^ which is considerably higher than the rate observed in our sample. These differences may be attributed to cultural practices, socioeconomic status, access to dental care services, and public awareness regarding oral hygiene. On the contrary, the similarity with the Ethiopian study may reflect shared social conditions and comparable educational infrastructure.

The findings of this study showed that 97% of students reported taking regular baths; daily bathing was reported by only 30.7% in summer and 5% in winter. This result closely aligns with findings from studies conducted in India (97.3%),^[14]^ Jordan (100%),^[21]^ and another study from India (100%).^[22]^ However, the rate observed in our study is higher than those reported in other parts of India (81%)^[11]^ and in Bangladesh (75.9%).^[23]^ These differences may be attributed to climatic conditions, seasonal water availability, cultural practices, and access to sanitation facilities.

The results of this study indicated that all participating students (100%) reported washing their hands before and after meals. This exceptionally high level of compliance is closely aligned with findings from studies conducted in India, where 98%,^[11]^ 96.4%,^[22]^ and 95.1%^[14]^ of students reported similar practices. However, the rate observed in our study exceeds those reported in other Indian studies, which found handwashing rates of 81%^[24]^ and 75.5%.^[25]^ This difference may be attributed to heightened public awareness regarding PH, particularly following the emergence of communicable diseases and the integration of practical hygiene education within school curricula in Kabul. Cultural norms, family reinforcement of healthy habits, and regular messaging through mass media may also play a significant role in influencing this behavior.

The findings of this study revealed that 99.5% of students regularly trimmed their fingernails. This figure is higher than those reported in studies conducted in India (89%),^[11]^ India (73.6%),^[14]^ and Jordan (82.8%).^[21]^ This difference may be attributed to several factors, including greater awareness of the role of personal cleanliness in disease prevention, consistent health education in school settings, and parental reinforcement of hygienic habits. In addition, the simplicity of the practice and easy access to personal grooming tools such as nail clippers may also contribute to this high rate of compliance.

The findings of this study have important implications for school health programs and public health policy in Afghanistan. Although the majority of students demonstrated good knowledge and hygiene practices, substantial gaps were identified, particularly in the use of soap for handwashing and the frequency of bathing and clothing changes. These findings highlight the need for targeted school-based hygiene education programs that emphasize practical skills and behavioral change rather than knowledge alone. Given the urban context of Kabul, where public schools often face overcrowding and limited sanitation resources, interventions should combine hygiene education with improvements in access to clean water, soap, and basic sanitation facilities to ensure sustainable behavioral change. Educational authorities and school administrators should integrate structured hygiene education into the school curriculum and provide adequate facilities, including access to clean water, soap, and sanitation infrastructure. Teachers can play a key role in reinforcing hygienic behaviors through regular health education sessions and role modeling. Furthermore, collaboration between the Ministry of Education and the Ministry of Public Health is essential for implementing comprehensive school health policies and monitoring systems. Community-based awareness campaigns involving parents may also help sustain hygiene practices outside the school environment. Strengthening these interventions could contribute to reducing the burden of hygiene-related infectious diseases among school-aged children in Afghanistan.

## 5. Limitations

This study has several limitations that should be considered when interpreting the findings. First, the research was conducted in a single high school (Habibia High School) in Kabul City, and all participants were male students. Therefore, the results may not be directly generalizable to female schools or other geographical regions of the country. Female students and students in rural schools may face different hygiene conditions due to limited access to water and sanitation, sociocultural influences, and variations in educational resources and school health programs.

Second, the study focused solely on high school students, leaving the hygiene knowledge and practices of students in lower grades unexamined. Third, although the questionnaire was adapted from validated tools used in previous studies, it may not have fully captured the local cultural and linguistic context. In addition, despite using a systematic sampling method, there may have been some selection bias during the distribution of questionnaires. Finally, as data were collected through self-reported measures, there is a possibility of response bias or overreporting of positive hygiene behaviors.

## 6. Conclusion

This study highlights a generally high level of knowledge about PH among school students in Kabul; however, a clear gap exists between knowledge and actual hygienic practices. While students demonstrated awareness of handwashing, nail trimming, and oral hygiene, practical adherence to proper hygiene behaviors, such as consistent use of soap and regular bathing, was inconsistent. Sociodemographic factors showed no significant association with hygiene behaviors, suggesting that environmental and behavioral barriers, including limited access to water, low motivation, and time constraints, play a larger role than age, grade, or parental education. These findings underscore the need for school-based interventions that combine structured health education with practical behavior-change strategies, such as supervised handwashing sessions and reinforcement of daily hygiene routines. Future research should explore underlying behavioral drivers and extend analyses to rural and underserved populations, where limited resources may further exacerbate gaps in hygiene practices. In addition, qualitative methods, such as in-depth interviews or focus groups, could provide richer insights into contextual factors influencing hygiene behaviors.

## Acknowledgments

We would like to express our sincere gratitude to the respected administration of Habibia High School for their cooperation throughout this study. We also deeply appreciate all the students of the high school at Habibia who willingly participated in our research with full consent.

## Author contributions

**Conceptualization:** Hafizullah Nikzad, Bashir Ahmad Qudrati, Shabir Ahmad Qudrati.

**Data curation:** Hafizullah Nikzad.

**Formal analysis:** Hafizullah Nikzad.

**Investigation:** Hafizullah Nikzad, Bashir Ahmad Qudrati, Shabir Ahmad Qudrati.

**Project administration:** Hafizullah Nikzad, Bashir Ahmad Qudrati, Shabir Ahmad Qudrati.

**Supervision:** Hafizullah Nikzad, Bashir Ahmad Qudrati.

**Validation:** Hafizullah Nikzad, Bashir Ahmad Qudrati, Mohammad Ali Reshad.

**Methodology:** Bashir Ahmad Qudrati, Mursal Maulawizada.

**Software:** Bashir Ahmad Qudrati, Shabir Ahmad Qudrati, Mursal Maulawizada

**Visualization:** Bashir Ahmad Qudrati.

**Writing – original draft:** Hafizullah Nikzad, Bashir Ahmad Qudrati.

**Writing – review & editing:** Hafizullah Nikzad, Bashir Ahmad Qudrati, Mohammad Ali Reshad, Shabir Ahmad Qudrati, Mursal Maulawizada.
